# Impacts from Waste Oyster Shell on the Durability and Biological Attachment of Recycled Aggregate Porous Concrete for Artificial Reef

**DOI:** 10.3390/ma15176117

**Published:** 2022-09-02

**Authors:** Jiafeng Kong, Songyuan Ni, Chen Guo, Mingxu Chen, Hongzhu Quan

**Affiliations:** College of Civil Engineering & Architecture, Qingdao Agricultural University, Qingdao 266109, China

**Keywords:** waste oyster shell, recycled aggregate porous concrete, durability, biological attachment, carbon dioxide emission

## Abstract

Poor biological attachment of artificial reef (AR) prepared by the recycled aggregate limit the application in the area of marine engineering. In this study, the waste oyster shell (WOS) was used as raw materials to prepare the recycled aggregate porous concrete (RAPC), the compressive strength, split tensile strength, chloride penetration resistance, freezing-thawing resistance, low temperature resistance, and the biological attachment were tested, aiming to improve the biological attachment and decrease carbon dioxide emission. The experiment results demonstrate that the use of WOS can decrease the compressive and split tensile strength, but the effect of designed porous structure on the mechanical strength is higher than that of WOS. To ensure the durability of RAPC, the contents of WOS should not exceed 20%. Additionally, the addition of WOS and designed porous structure are beneficial to biological attachment. However, the porous structure of RAPC only improves biological attachment in the short term, and the reverse phenomenon is true in the long term. As the partial replacement of cement with WOS is 40%, the total carbon dioxide emission decreases by about 52%. In conclusion, the use of WOS in the RAPC is an eco-friendly method in the artificial reef (AR) with improved ecological attachment and reduced carbon dioxide emission.

## 1. Introduction

Waste management is one of the most worldwide problems, such as construction and demolition waste (CDW) and aquacultural waste (AW), leading to lots of economic losses and environmental threats caused by their heaps [1,2], and systematic consumption is essential. Recently, CDW was used to decrease carbon dioxide emission in the building material, which aimed to provide effective measures to solve the greenhouse problem, and the study of concrete is shifting toward sustainability [3,4]. The production of CDW reached 400 million per year in China [5] and most of them were utilized to prepare the recycled aggregate concrete [6,7,8], especially on the low strength engineering, such as plant concrete and artificial reefs concrete. For instance, Li et al. [9] used the recycled aggregate to produce planting concrete, and they discovered that the frost resistance is enhanced by 12.4%, and the drying shrinkage is reduced by 20.5%. Kong et al. [10] used WOS and recycled aggregate (RA) to prepare the porous ecological concrete toward AR, and they found that the interconnected porosity was affected less in ecological concrete, and the alkalinity of leachate becomes close to seawater.

In China, as a kind of AW, the output of WOS can reach 12–15 million tons per year [11]; some studies related to the concrete with WOS have been conducted. Yang et al. [12] used oyster shells (OS) to replace fine aggregate with three substitution rates (SR) of 5%, 10%, and 20%, and they found that the addition of OS did not reduce the compressive strength cured for 28 d, and the compressive strength was improved as the SR increased. Yoon et al. [13] investigated the mechanical and chemical properties of crushed OS, and they found that the compressive strength presented no significant reduction with the replacement rate of OS reaching 40%.

AR is a man-made structure in the sea, aiming to provide suitable habitats for marine life, enhance fisheries, and capture biological carbon [14,15]. Concrete is the widest raw material of AR, but the use of natural material in producing concrete presents resource depletion and poor economy [16]. Meanwhile, in AR prepared with concrete there exist some problems, such as poor durability and biological attachment [17,18]. Recently, some related studies demonstrated poor biological attachment when the AR was placed in the sea. For instance, Ly et al. [19] produced AR by 3D printing using geopolymer, cement and recycled sand to assess micro- and macro-organisms and the durability after 1, 3 and 6 months, and they found that cement was better than geopolymer binders in 3D printed concrete for AR. Chlayon et al. [20] investigated combined protective and negative effects of calcareous-based sessile invertebrates and root-spreading botanic life forms, and they found that surface treatment exhibited decent quantities of barnacle and oyster while restricting the amount of algae settled. Recently, porous concrete was used to prepare AR. Porous concrete presents porous structure, which may affect strength and durability [21,22,23]. Some studies have proved the multiple environmental benefits [24,25], including increasing planting properties [26], reducing water and soil pollution [27].

However, AR prepared with porous concrete presents poor biological attachment. Therefore, in this study, the WOS was used as raw materials to prepare the RAPC, aiming to improve the biological attachment and decrease carbon dioxide emission. To ensure the durability of RAPC, the chloride penetration resistance, freezing–thawing resistance, and low temperature resistance were tested. Furthermore, marine ecology was used to investigate the species distribution of attached organisms and the stability of the ecosystem. Meanwhile, the use of WOS plays an important role in reducing carbon dioxide emission.

## 2. Materials and Methods

### 2.1. Raw Materials and Preparation Procedures

Ordinary Portland cement (OPC, 42.5 R, Shanshui company, Qingdao, China) was used as the cementitious materials and WOS (collected from Qingdao, China) was used as the inert filler, with the physical and chemical properties of these in Table 1 and Table 2. The water reducing agent (Aliphatic, Sobute New Materials company, Beijing, China), with a water reducing rate of 25%, was used to increase the flowability of fresh paste. The particle size of recycled aggregate (RA, Qingdao Lvfan company, CHN) was in the range of 5~20 mm, and its physical properties were shown in Table 3. The absolute volume method was used to obtain the mix design [28,29]. WOS was used to replace cement in the designed concrete, and the replacement rates were 0%, 20% and 40% (by mass), respectively. The porosity of RAPC was designed at 10%, and the control group is compacting recycled aggregate concrete (CR). The detailed mix design was shown in Table 4. The specimens of 3 to 6 were made for the test.

**Figure 1:**
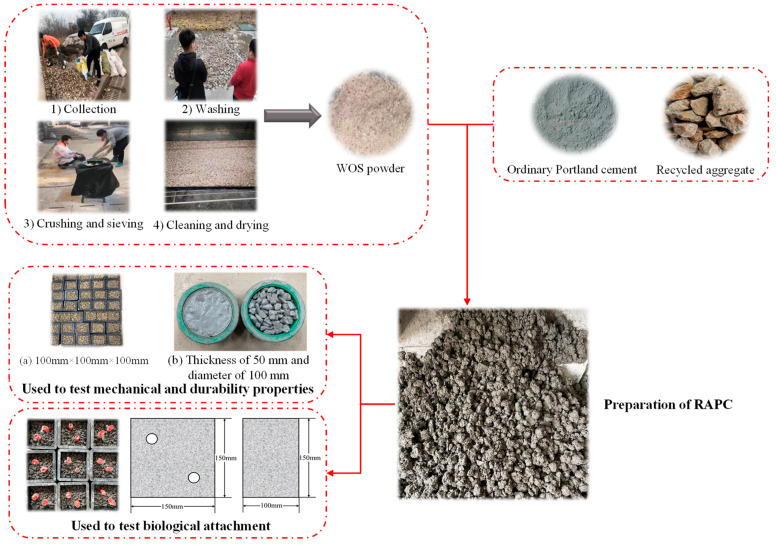
The flow chart of prepared specimens.

The flow chart of prepared specimens was shown in Figure 1, and the detailed preparation procedures were as follows:

(1) The WOS powder and cement were firstly mixed in the concrete mixer for 30s;
(2) Water reducing agent was dissolved in the water and then to add and mix in the concrete mixer for 60s;
(3) The recycled aggregate was mixed with the above cement paste for 90s to get the fresh concrete;
(4) The prepared RAPC was cast into the molds and subsequently removed from the molds after 1d;
(5) The specimens were cured at a temperature of 20 ± 2 °C and humidity of 95% RH.

### 2.2. Test Methods

#### 2.2.1. Mechanical Properties

According to GB/T 50081–2019 [30], the compressive strength and splitting tensile strength of concrete cured for 28 d were tested using a testing machine (YA 2000, Cangzhou Zerui company, Cangzhou, China), with the loading speed set at 0.5 kN/s. The tension-compression ratio was used to evaluate the brittleness of concrete, which was calculated by Equation (1).

(1) $$R_{T\text{-}S}=\frac{f_{s}}{f_{t}}$$

where ***R_T-S_*** is the tension-compression ratio of specimen, dimensionless; ***f_s_*** is the compressive strength of specimen, MPa; ***f_t_*** is the split tensile strength of specimen, MPa.

#### 2.2.2. Durability

##### Chloride Penetration Resistance

According to GB/T 50082–2009 [31] and ASTM C1202 [32], two methods, including rapid chloride irons migration (RCM) and rapid chloride permeability (RCP), were used for testing the chloride penetration resistance. The non-steady-state chloride migration coefficient, and electric flux, calculated by Equations (2) and (3), respectively, were used to evaluate the chloride penetration resistance.

(2) $$D_{RCM}=\frac{0.0239\times \left(273+T\right)}{\left(U-2\right)t}\left(X_{d}-0.0238\times \sqrt{\frac{\left(273+T\right)\times L\times X_{d}}{U-2}}\right)$$

where, ***D_RCM_*** is the non-steady-state chloride migration coefficient, ×10^−12^ m^2^/s; ***T*** is average initial and final temperatures in anolyte and catholyte solutions, °C; ***L*** is specimen thickness, mm; ***U*** is applied voltage, ***V***; ***t*** is test duration time, ***h***; ***X_d_*** is average penetration depth, mm.

(3) $$Q_{s}=900\times \left(I_{0}+2\times I_{30}+2\times I_{60}+\ldots +2\times I_{300}+2\times I_{330}+2\times I_{360}\right)$$

where, ***Q_s_*** is electric flux, C; ***I***_0_ is initial current, A; ***I_t_*** is current at t min, A;

##### Freezing-Thawing Resistance

According to GB/T 50082–2009 [31], the slow freezing method was used to test the freezing–thawing resistance. The testing machine (TDS-300, Donghua Examination apparatus company, CHN) was adopted. The average mass loss rate and strength loss rate were calculated by Equations (4) and (5), respectively, to evaluate freezing–thawing resistance.

$$D_{mi}=\frac{\left(D_{0i}-D_{ni}\right)}{D_{0i}}\times 100$$

(4) $$D_{m}=\frac{\sum_{i=1}^{3}D_{mi}}{3}\times 100$$

where, ***D_mi_*** is mass loss rate, %; ***D***_0***i***_ is initial weight of specimen, g; ***D_ni_*** is the weight of the specimen under n times freezing–thawing cycles, g; ***D_m_*** is the average mass loss rate, %.

(5) $$D_{s}=\frac{W_{c0}-W_{cn}}{W_{c0}}\times 100$$

where ***D_s_*** is strength loss rate, %; ***W_c_***_0_ is the compressive strength of specimen cured in the standard curing chamber, MPa; ***W_cn_*** is the compressive strength of specimen on freezing-thawing cycles after n times, MPa.

##### Low Temperature Resistance

The low temperature testing machine (STDW-40D, temperature accuracy reaches 0.1 °C, Shanghai Civil & Road company, Shanghai, China) was used for testing the resistance of concrete to low temperature. The test specimens were stored in testing machine after being cured for 28 d, and the temperature was set to 2 °C. The specimens were taken and tested for compressive strength at the end of 30 d, 60 d, and 90 d, respectively. The coefficient of compressive strength is calculated by Equation (6).

(6) $$K_{f}=\frac{L_{cn}}{L_{c0}}\times 100$$

where ***K_f_*** is the coefficient of compressive strength, %; ***L_cn_*** is the compressive strength of the specimen on low temperature after *n* days, MPa; ***L_c_***_0_ is the compressive strength of specimen cured in the standard curing chamber, MPa.

#### 2.2.3. Biological Attachment

##### Testing Program

**Figure 2:**
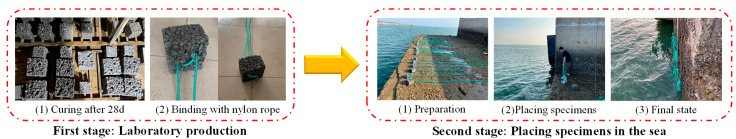
The flow chart of placed specimens.

The prepared concrete was immersed in the sea located in Rizhao, China. The depth of immersion was 2 m below the surface of the seawater at low tide level, aiming to reduce the effects of environmental changes and ensure the immersed condition, as shown in Figure 2. Subsequently, specimens were tested after 1 month, 3 months and 6 months, respectively.

The specimens were operated as follows. Firstly, specimens were re-floated and photos taken, then attached organisms were obtained using a scraping tool from the area of 100 mm × 150 mm. Subsequently, the attached organisms were brought to the laboratory using a glass bottle and adding 10% formaldehyde solution (50 mL) to fix with the organisms. The test program for biological identification was as follows. Firstly, the specimens in the glass bottle were mixed and then dropped onto the glass slide using a dropper. Inverted microscopes (Axio vert A1, ZEISS, Oberkochen, Germany) were used to observe the specimens and check into the China Species Library. Finally, the observed specimens were re-collected and dried until their weight no longer changed (temperature was 105 °C ± 10 °C).

##### Assessment Methods of Biological Attachment

The biological attachment was evaluated by two methods: (1) the visual assessment using a macrograph; (2) biological identification. Additionally, the biological attachment index, Shannon-Wiener index and Pielou evenness index, calculated by Equations (7)–(9), respectively, provide complementary data to evaluate the biological attachment.

(7) $$P=\frac{m}{s}$$

where, ***P*** is the biological attachment index, kg/m^2^; ***m*** is the dry weight of attached organisms, kg; ***s*** is taken specimens area, m^2^.

(8) $$H^{\prime }=-\sum_{i=1}^{s}P_{i}\log_{2}P_{i}$$

(9) $$J=\frac{H^{\prime }}{\log_{2}S}$$

where, $H^{\prime }$ is the Shannon-Wiener index, dimensionless; ***J*** is the Pielou evenness index, dimensionless; ***S*** is species number of attached organisms, number/50 mL; ***P_i_*** is the particular organism to the total organism ratio, dimensionless.

#### 2.2.4. Carbon Dioxide Emission

This study evaluated the carbon dioxide emission of concrete within the boundary, including the preparation and transportation of raw materials, and the carbon dioxide sequestration of biological attachment. The function unit was defined as “1 m^3^ concrete in this process”. According to related standards and research, the carbon dioxide emission factor of mainly energy and composition in concrete were shown in Table 5 and Table 6. However, the carbon dioxide emission factor of WOS did not present available data and was calculated by integrating the energy consumption during crushing, screening, and transportation, as shown in Table 7. The total carbon dioxide emission was calculated by Equations (10)–(13). The mainly biological attachments were shellfish after 6 months in the sea. Therefore, the hypothetical scenario is the shellfish accounted for 95% and the related index was built. The carbon dioxide sequestration factor of shellfish in China is 0.1157 kg CO_2_-eq/t [33].

(10) $$C=C_{r}-C_{wos}-C_{bio}$$

where, ***C*** is the total carbon dioxide emission of concrete, kg CO_2_-eq; ***C_r_*** is the carbon dioxide emission of raw materials, kg CO_2_-eq; ***C_wos_*** is the carbon dioxide sequestration of WOS as raw material, kg CO_2_-eq; ***C_bio_*** is the carbon dioxide sequestration of biological attachment, kg CO_2_-Equation

(11) $$C_{r}=\sum^{\;}F_{i}\times m_{i}$$

where, ***F_i_*** is the carbon dioxide emission factor of ***i*** type of raw material, kg CO_2_-eq/t; ***m_i_*** is the weight of ***i*** type of raw material in 1 m^3^ concrete, t.

(12) $$C_{wos}=E_{wos}\times m_{wos}$$

where, ***E_wos_*** is the carbon dioxide sequestration factor of WOS, kg CO_2_-eq/t; ***m_wos_*** is the weight of WOS as raw material in 1 m^3^ concrete, t.

(13) $$C_{bio}=E_{bio}\times \varepsilon \times m_{bio}$$

where, ***E_bio_*** is the carbon dioxide sequestration factor of biological attachment, kg CO_2_-eq/t; *ε* is 0.95 meaning the related index, dimensionless; ***m_bio_*** is the dry weight of biological attachment in 1 m^3^ concrete, t.

## 3. Results and Discussion

### 3.1. Mechanical Properties

**Figure 3:**
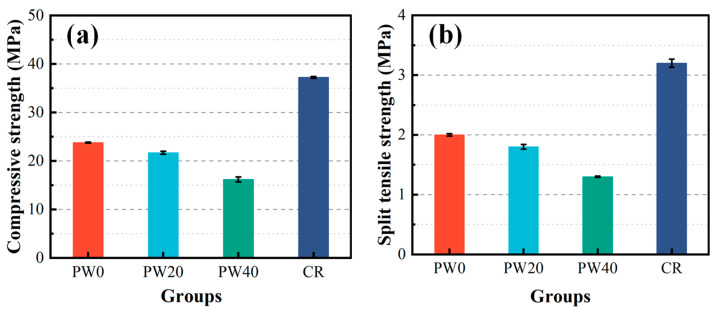
The compressive strength and split tensile strength of concrete. (**a**) compressive strength. (**b**) split tensile strength.

**Figure 4:**
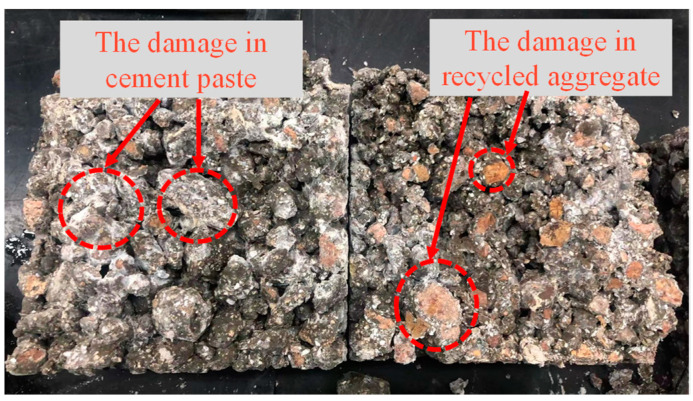
The interfacial broken state of RAPC adding WOS.

**Figure 5:**
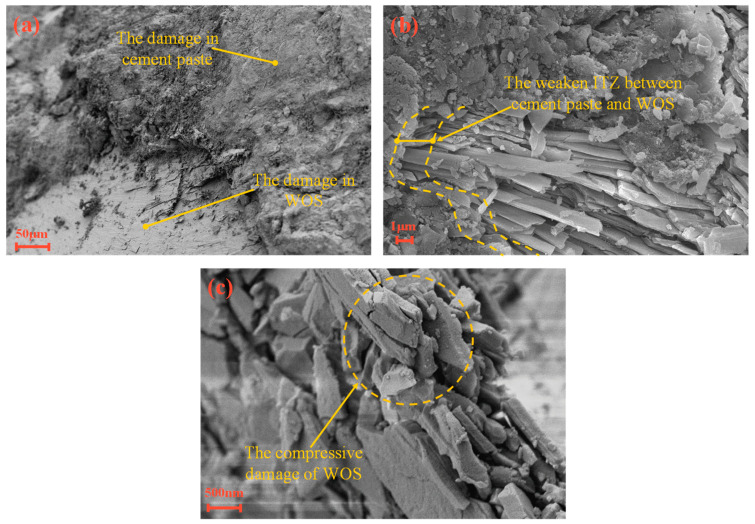
The micro-structures of RAC: (**a**) The hardened paste is damaged. (**b**) The ITZ makes it weaken due to broken WOS. (**c**) The smooth surface of WOS is broken.

The mechanical properties of concrete are evaluated by compressive strength and split tensile strength, as shown in Figure 3. The compressive strength and split tensile strength decrease from 9% to 32% and from 10% to 35%, respectively, when the partial replacement of cement with WOS is 0, 20, and 40%. The reason is that the addition of WOS with smooth surface in the RAPC causes the interfacial transition zone (ITZ) to be weak, as shown in Figure 4 and Figure 5a. Meanwhile, the addition of WOS as inert material reduces the content of cementitious material, which decreases the strength of hardened cement paste, as shown in Figure 5b,c. However, compared with CR, the compressive strength and split tensile strength of RAPC decrease by 36% and 38%, respectively. The reason is that the strength of RAPC relies on the bonding region [38], which is weakened due to the high water absorption and low strength of RA.

Furthermore, due to the impact effect of sea waves, the AR will suffer from vertical compressive, horizontal split tensile and shear stresses in the seawater. Therefore, the brittleness of concrete is investigated. The tension-compression ratio is used to assess the brittleness of concrete, as shown in Table 8. The lower tension-compression ratio means greater brittleness and smaller toughness. As the partial replacement of cement with WOS is 0, 20, and 40%, the reduction degree of tension-compression ratio is in the range of 1~5%. Compared with CR, the tension-compression ratio of RAPC decreases by 2%.

### 3.2. Durability

#### 3.2.1. Chloride Penetration Resistance

**Figure 6:**
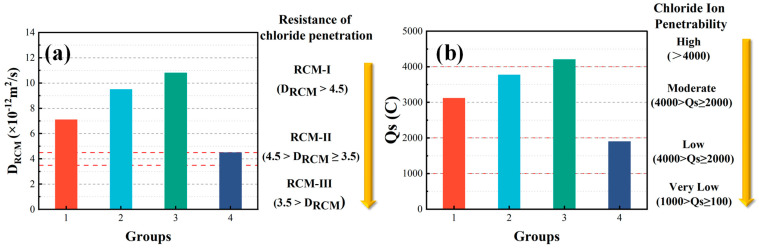
The test results of chloride penetration resistance: (**a**) RCM; (**b**) RCP.

**Figure 7:**
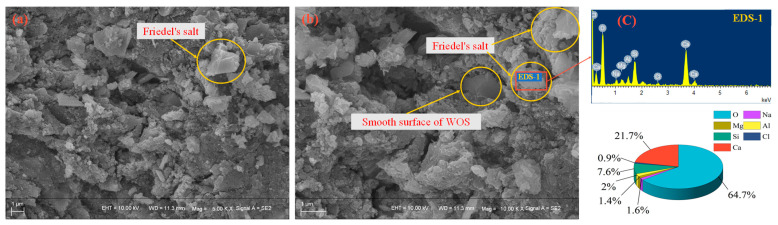
The micro-structures of concrete: (**a**,**b**) Friedel’s salt is formed; (**c**) EDS.

The non-steady state chloride migration coefficient and electric flux are used to evaluate the chloride penetration resistance, as shown in Figure 6. As the partial replacement of cement with WOS is 0, 20, and 40%, the non-steady state chloride migration coefficient and electric flux decrease by 25~34% and 17~26%, respectively. The reason is that the smooth surface of WOS prevents C-S-H gel and other hydration product from attaching [39], as shown in Figure 7b. Further, the addition of WOS leads to higher porosity in hardened cement paste, which increases the permeability of concrete. Meanwhile, the lower pH values provide a greater capacity that the AFm phases of cement hydration product contact with dissolved chloride ions to form Friedel’s salt [40], as shown in Figure 7c. The reaction (14) was as follows:

(14) $$3\mathrm{CaO}\;{\mathrm{Al}}_{2}O_{3}\;{\mathrm{CaSO}}_{4}\;12H_{2}O+2{\mathrm{Cl}}^{-}\to 3\mathrm{CaO}\;{\mathrm{Al}}_{2}O_{3}\;{\mathrm{CaCl}}_{2}\;10H_{2}O+{\mathrm{SO}}_{4}^{2-}+2H_{2}O$$

Compared with CR, the non-steady-state chloride migration coefficient and electric flux of RAPC decrease by about 37% and 39%, respectively. The reason is that RA with micro cracks and high water absorption will lead to a weakened ITZ [41]. Therefore, the concrete is easily penetrated by chloride iron due to the ITZ with higher porosity.

#### 3.2.2. Freezing–Thawing Resistance

**Figure 8:**
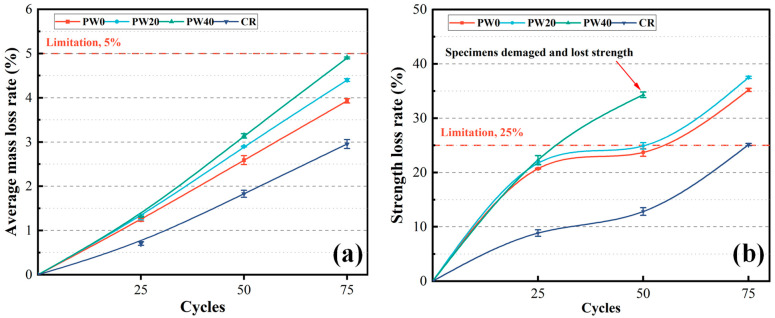
The test results of freezing–thawing resistance: (**a**) Average mass loss rate. (**b**) Strength loss rate.

**Figure 9:**
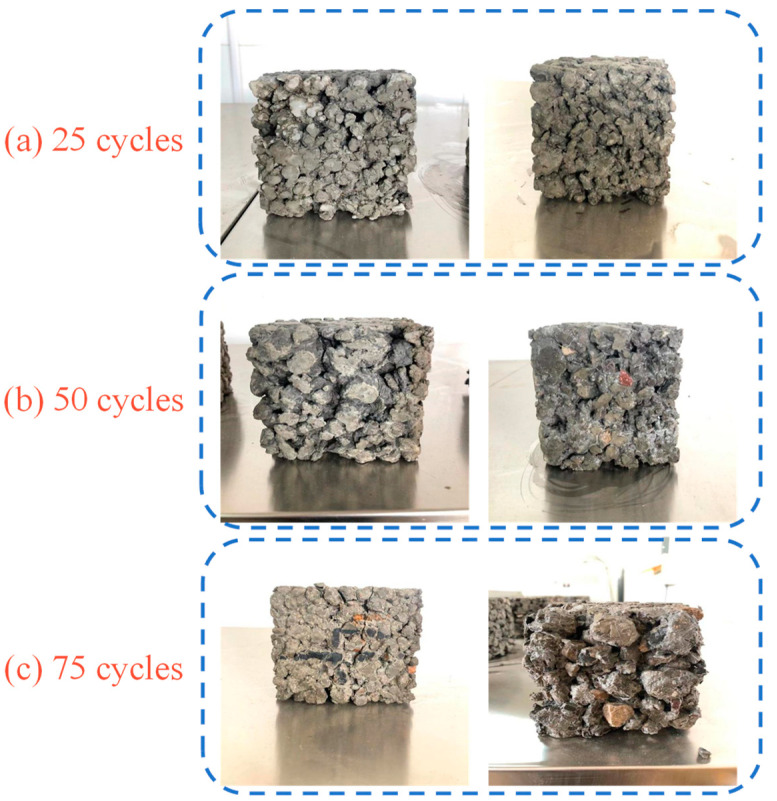
The morphologies of specimens after 25, 50, and 75 freezing–thawing cycles.

The freezing–thawing resistance of concrete is evaluated by the average mass loss rate and the strength loss rate, as shown in Figure 8. After 25 times of the freezing–thawing cycles, as the partial replacement of cement with WOS is 0, 20, and 40%, the average mass loss rate and the strength loss rate increase 6% and 8%, respectively, and the surface of concrete changes less, as shown in Figure 9a. However, compared with CR, the average mass loss rate and the strength loss rate of RAPC increase by about 43% and 57%, respectively. The reason is that RAPC presents higher permeability, and the water easily infiltrates inside. Meanwhile, the small pores in RA easily reach water saturation. At the same rate of temperature drop, the hydraulic pressure and permeation pressure are generated inside the concrete owing to the water freeze in large cavities and salt concentration differences in the pore fluid, respectively [42]. During this process, the cement paste and ITZ are gradually broken, and the bonding region of RAPC presents stress concentration and damage, which the strength of RAPC depends on the bonding region. As a result, the strength loss rate decreased significantly.

After 50 times of the freezing–thawing cycles, as the replacement rate of WOS is 20%, the average mass loss rate and the strength loss rate of RAPC increase by 12% and 5%. The weight and strength loss rate of RAPC meet the requirements of GB/T 50082-2009 [31]. However, the average mass loss rate and the strength loss rate of RAPC increase by 21% and 45%, respectively, as the replacement rate of WOS reached 40%. At this time, the surface of concrete occurs to crack and scaling, as shown in Figure 9b. The reason is that the addition of WOS increases the porosity of hardened cement paste, including gel pores, capillary cavities, and large cavities. WOS presents a porous structure and smooth surface, and ITZ is increased around the WOS, whose smooth surface prevents the attachment of C-S-H gel, as shown in Figure 5c. Furthermore, the use of WOS decreases the content of cement, which led to the reduction of hydration product. However, compared with CR, the average mass loss rate and the strength loss rate of RAPC increase by 29% and 46%, respectively.

After 75 times of the freezing–thawing cycles, as the partial replacement of cement with WOS is 0, 20, and 40%, the average mass loss rate and the strength loss rate increase by 12~25% and 7%, respectively. The specimen of PW40 has been broken and lost strength, as shown in Figure 9c. Compared with CR, the average mass loss rate and the strength loss rate of RAPC increase by about 25% and 29%, respectively. At this time, the surface of concrete presents heavy cracks, scaling, and even visible cracking.

#### 3.2.3. Low Temperature Resistance

**Figure 10:**
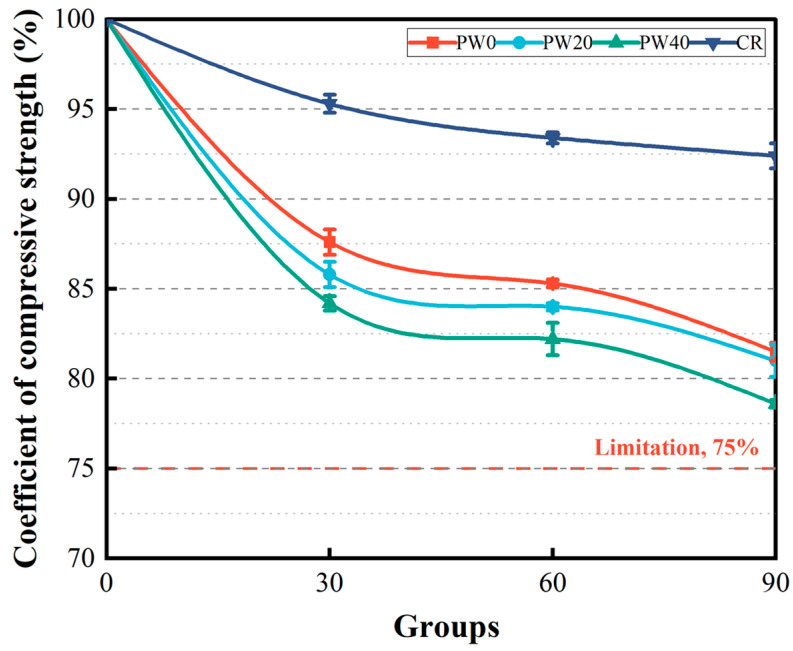
The coefficient of compressive strength of concrete after 30, 60, and 90 cycles.

The coefficient of compressive strength is used to evaluate the low temperature resistance of concrete, as shown in Figure 10. As the replacement rate increases from 0% to 40%, the coefficient of compressive strength decreases by 2%, 2%, and 1~3%, respectively, when RAPC was tested on 30, 60, and 90 low temperature cycles. The reason is that the increased porosity led to the decreased strength of cement paste. Meanwhile, low temperature prevents further hydration of cement and strength from increasing. However, compared with CR, the coefficient of compressive strength of RAPC decreases by 8%, 9%, and 12%, respectively, when RAPC was tested on 30, 60, and 90 low temperature cycles. The reason is that the addition of WOS decreases the strength of bonding region. All specimens meet requirements in GB/T 50082-2009 [31] and can bear the strength loss of a temperature change in the sea.

### 3.3. Biological Attachments Analysis

#### 3.3.1. Biological Attachment Density

**Figure 11:**
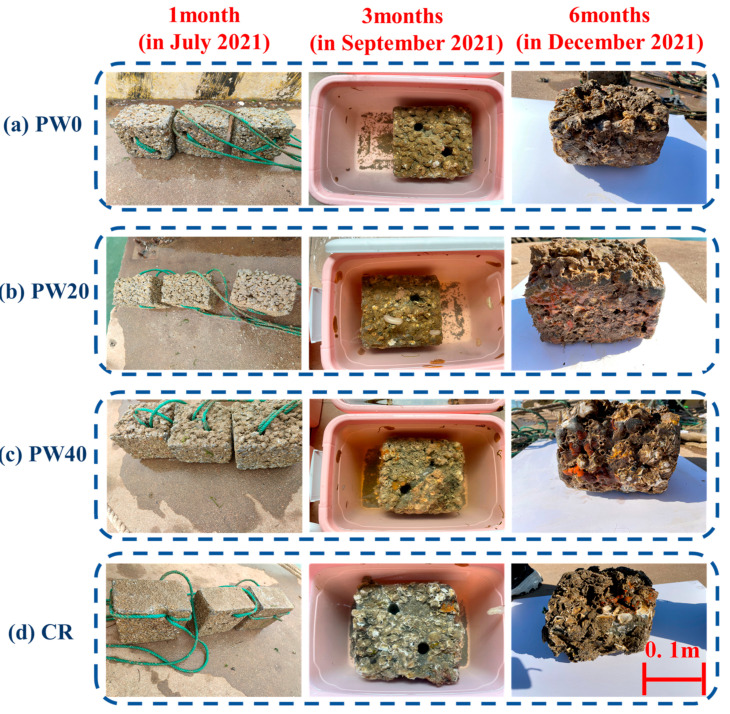
Macrograph of the growth state of the attached organisms.

The state of all specimens is observed differently, and the macrograph of the growth state for attached organisms is as shown in Figure 11. In 1 month, as the partial replacement of cement with WOS is 0, 20, and 40%, the biological attachment distinguished is increased. The reason is that the decreased cement content leads to the reduction of pH value on the surface of concrete [43]. Meanwhile, the nutritional contents in the WOS provide the basic conditions for the attachment of benthic algae and protozoa, which are crucial for the establishment of the microbial communities and succession, as well asthe large organisms [44]. In 3 months and 6 months, the weight of biological attachment increases.

Compared with CR, RAPC is beneficial to the biological attachment in 1 month due to the bigger specific surface area and rougher surface. As marine life is carried by seawater across the RAPC surface, some organic small molecules are rapidly accumulated, such as polysaccharides, proteins, etc., and biofilm is formed on the surface [45,46]. Subsequently, the biofilm continuing to adsorb foreign cells relied on sponge-like extracellular matrix, which provides basic conditions for the attachment of large organisms [47]. However, in 3 months and 6 months, the advantage of biological attachment in RAPC disappear due to the pore being filled by attached organisms.

#### 3.3.2. Assessment of Attached Biological Attachment

**Figure 12:**
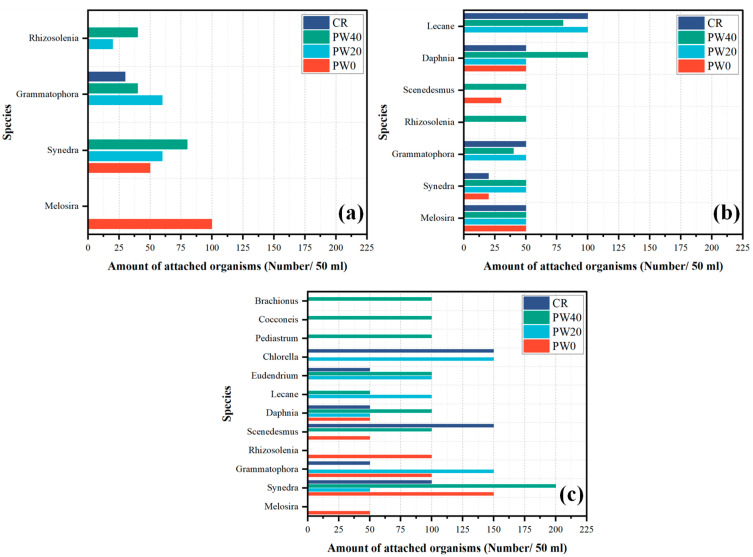
The species of biological identification at 1, 3, and 6 months: (**a**) 1 month; (**b**) 3 months; (**c**) 6 months.

**Figure 13:**
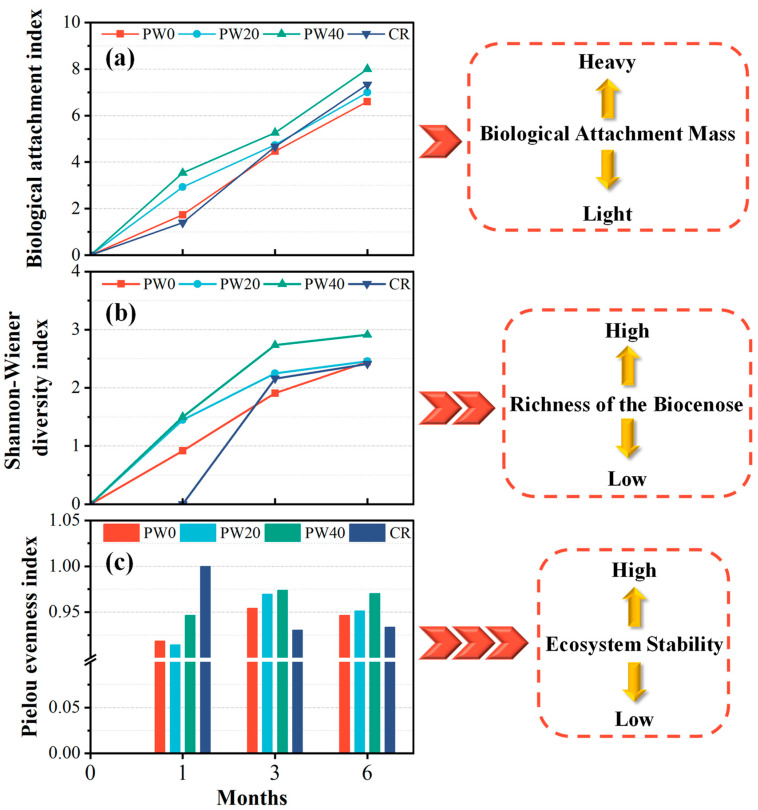
The three indexes at 1, 3, and 6 months: (**a**) biological attachment index; (**b**) Shannon-Wiener index 3 months; (**c**) Pielou evenness index.

Biological identification and three indexes at different times were used to further assess the biological attachment. The species of biological identification and three indexes at 1, 3, and 6 months are shown in Figure 12 and Figure 13, respectively. In 1 month, as the partial replacement of cement with WOS is 0, 20, and 40%, the biological attachment index increases by 69.2% and 103.8%, respectively. Compared with CR, the biological attachment index of RAPC increases by 23.8%, indicating that the porous structure increased the attached effect in an early stage. The species of biological identification are three for PW20 and PW40, two for PW0 and one for CR, respectively. The order of the Shannon-Wiener index is PW40 > PW20 > PW0 > CR, showing that the richness of the biocenose is the biggest for PW40. The order of the Pielou evenness index is CR > PW40 > PW0 > PW20. However, only one species was attached for CR, and the Pielou evenness index is 1, which lacks representation. Besides CR, the Pielou evenness index of PW40 is the biggest, indicating that PW40 not only presents higher richness of the biocenose, but also a more stable ecosystem owing to small differences in the group. In 3 months, as the partial replacement of cement with WOS is 0, 20, and 40%, the biological attachment index increases by 6% and 17.9%, respectively. Compared with CR, the biological attachment index of RAPC decreases by 4.5%. The species of biological identification are seven for PW40, and five for PW20, CR, and PW20, respectively. The order of the Shannon-Wiener index is PW40 > PW20 > CR > PW0, which shows the highest richness of the biocenose of PW40. The order of the Pielou evenness index is PW40 > PW20 > PW0 > CR, indicating that PW40 present smaller difference in each group. Combined with Shannon-Wiener index, the attached effect of PW40 is better than others. In 6 months, as the partial replacement of cement with WOS is 0, 20, and 40%, the biological attachment index increases by 6.1% and 21.2%, respectively. Compared with CR, the biological attachment index of RAPC decreases by 4.5%. The species of biological identification are seven for PW40, and six for PW20, PW0, and CR, respectively. The order of the Shannon-Wiener index is PW40 > PW20 > PW0 > CR, but the differences between PW20, PW0, and CR are not obvious, presenting excellent richness of the biocenose. The order of the Pielou evenness index is PW40 > PW20 > PW0 > CR, presenting the most stable ecosystem of PW40.

In summary, the addition of WOS and design of RAPC are effective methods to increase the biological attachment of concrete toward the use of AR. The biological attachment, the richness of the biocenose and the stability of the ecosystem is improved. However, compared with CR, the improvement of biological attachment in the RAPC only exists in the short term, and a negative effect is presented in the long term due to the high pH value on the surface of concrete.

### 3.4. Carbon Dioxide Emission

**Figure 14:**
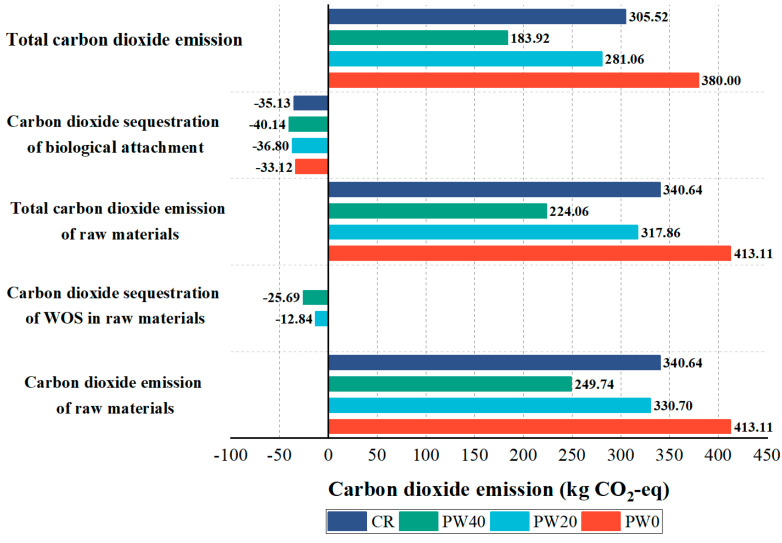
The carbon dioxide emission for concrete at different stages.

The total carbon dioxide emission is used to evaluate the carbon reduction effect, as shown in Figure 14. The highest carbon dioxide emission in the raw material of concrete is cement accounting for more than 98%. As the partial replacement of cement with WOS is 0, 20, and 40%, the total carbon dioxide emission decreases by 26~52%; the three main reasons are below, firstly, the reduction of cement is the essential method due to the high carbon dioxide emission in the raw materials. Secondly, the formed oyster shell will sequester carbon, and the use of WOS is a feasible method to achieve carbon dioxide sequestration. Thirdly, the increasing biological attachment can further sequester carbon. However, the total carbon dioxide emission of RAPC increases 24% more than CR. The RAPC does not achieve more effective carbon reduction than CR due to the high content of cement and the limited effect on biological attachment in the long term.

In summary, the use of WOS is a potential method to reduce carbon dioxide emission. Further, the designed porous structure should be paid attention to and combined with practical application. Although its rough surface can increase biologically in the short term, a high carbon dioxide emission is adverse to the environment.

## 4. Conclusions

In this study, the WOS was used as the raw materials to prepare the RAPC, aiming to improve the biological attachment and decrease carbon dioxide emission. In conclusion, the use of WOS in the RAPC is an eco-friendly method in the AR with improved ecological attachment and reduced carbon dioxide emission. The main conclusions are highlighted below:

(1) The addition of WOS can decrease the compressive and split tensile strength, but the effect of designed porous structure on the mechanical strength is higher than that of WOS.
(2) To ensure the durability of RAPC, the contents of WOS should not exceed 20%.
(3) The addition of WOS and designed porous structures are beneficial to the improvement of biological attachment. However, the porous structure of RAPC only improves biological attachment in the short term, and the reverse phenomenon is true in the long term.
(4) As the partial replacement of cement with WOS is 40%, the total carbon dioxide emission decreases by about 52%.

## Figures and Tables

**Table 1:** The physical properties of OPC and WOS.

Type	Density (kg/m^3^)	Specific Surface Area (m^2^/kg)	Setting Time (min)		Flexural Strength (MPa)		Compressive Strength (MPa)	
			Initial Setting Time	Final Setting Time	3d	28d	3d	28d
OPC	3100	342	182	251	4.7	7.5	21.8	47.6
WOS	2850	313	-	-	-	-	-	-

**Table 2:** The chemical properties of OPC and WOS.

Material	SiO_2_	AI_2_O_3_	Fe_2_O_3_	CaO	MgO	SO_3_	Na_2_O	LOI
OPC	21.31	5.68	3.54	61.72	1.3	2.58	2.89	1.05
WOS	1.05	0.21	0.27	95.06	0.67	0.56	1.28	0.91

**Table 3:** Physical properties of recycled aggregate.

Material	Aggregate Size (mm)	Stacking Density (kg/m^3^)	Apparent Density (kg/m^3^)	Water Absorption (%)	crushing Index (%)
RA	5-20	1420	2623	5.2	14

**Table 4:** The mix design of 1 m^3^ concrete.

Specimen	Replacement Rate (%)	Unit Weight (kg)					
		Cement	Water	RA	WOS	Water Reducer	Sand
PW0	0	559	168	1598	-	0.6	-
PW20	20	445	167	1598	111	1.3	-
PW40	40	333	167	1598	222	1.7	-
CR	0	461	175	1252	-	1.4	512

**Table 5:** Carbon dioxide emissions factor of main energy.

Energy	Total Carbon Dioxide Emissions (kg CO_2_-eq)	Note
Electricity (1 kW·h)	1.195	The data is derived from the IPCC and literature [34].
Coal (1 kg)	2.618	It is obtained by data conversion from the literature [35].
Diesel (1 L)	3.178	It is obtained by data conversion from the literature [35].

**Table 6:** Carbon dioxide emissions factor of composition in concrete.

Species	Total Carbon Dioxide Emissions (kg CO_2_-eq/t)	Note
Cement	735	The data is derived from GB/T 51,366 [36].
Water	0.347	It is obtained by data conversion from the literature [37].
Water reducer	30.39	It is obtained by data conversion from the literature [37].
RA	1.36	It is obtained by data conversion from the literature [37].
Sand	2.51	The data is derived from GB/T 51,366 [36].

**Table 7:** Carbon dioxide emissions factor of 1t WOS.

Species	Content		Note
WOS	**Processing WOS**		
	Crushing (kW·h)	4.41	The power of crusher is 3 kW, and the efficiency of machine is 680 kg/h.
	Sieving (kW·h)	1.6	The power of screening equipment is 8 kW, and the machining efficiency is 5000 kg/h.
	Carbon dioxide emissions factor (kg CO_2_-eq/t)	7.18	Calculated by Table 5.
	**Transporting WOS**		
	Distance (km)	15	Transportation distance from collection place to laboratory.
	Carbon dioxide emissions factor (kg CO_2_-eq/t)	5.01	Diesel truck is used to transport, and its carbon dioxide emissions factor is 0.334 [kg CO_2_-eq/(t·km)] [36].
	**Total Carbon dioxide emissions factor (kg CO_2_-eq/t)**	**12.19**	-

**Table 8:** Tension-compression ratio of concrete.

Groups	PW0	PW20	PW40	CR
R_T-S_	0.084	0.083	0.080	0.086
